# Impact of Hydroxychloroquine on Antibody Responses to the SARS-CoV-2 Coronavirus

**DOI:** 10.3389/fimmu.2020.01739

**Published:** 2020-10-30

**Authors:** Isabel Kinney Ferreira de Miranda Santos, Carlos Henrique Nery Costa

**Affiliations:** ^1^Ribeirão Preto School of Medicine, University of São Paulo, São Paulo, Brazil; ^2^Nathan Portela Institute of Tropical Medicine, Federal University of Piauí, Teresina, Brazil

**Keywords:** COVID-19, Sars-CoV-2, coronavirus, antibodies, hydroxychloroquine

- Humoral immunity is a crucial aspect in mitigating the COVID-19 pandemic.
- Hydroxychloroquine and chloroquine are lysosomotropic drugs that affect antigen-presenting pathways and B-cell activation.
- Chloroquine inhibits antibody responses to vaccines, but reports about this effect apparently have not been called to the attention of investigators in the field of COVID-19.
- Studies on immunity to Sars-CoV-2 must take into account treatment regimens for COVID-19.

## Introduction

Recent large observational studies indicate that hydroxychloroquine (HY) does not affect outcomes of patients hospitalized with COVID-19 (1, 2) and may even be harmful (3). Results of double-blind, randomized studies to assess efficacy of HY more rigorously are still not available. In spite of these facts, officials are currently advocating use of hydroxychloroquine (HY) for treatment and even prevention of COVID-19. In view of this situation and of the importance of correct interpretation of antibody profiles for planning preventive measures for COVID-19, we would like to bring the attention of readers to studies that raise concerns about the possible impact of HY upon antibody responses to SARS-CoV-2.

## Impact of Chloroquine and Derivatives on Responses to Vaccines and Antigen Presentation

In 1986, Pappaionaou et al. (4) recalled the tragic story of a Peace Corps worker in Kenya who succumbed to rabies after being bitten by her rabies-infected pet dog despite having received a full regimen of human diploid-cells rabies vaccine 6 months prior to the bite. The subject had been vaccinated while receiving chloroquine as prophylaxis for malaria. Prompted by this finding, Pappaionaou et al. carried out a randomized controlled trial that showed that chloroquine suppressed antibody responses to the rabies vaccine (4). Subsequently, Fryauff et al. demonstrated a similar effect of chloroquine, but not of primaquine, on antibody responses to tetanus and diphtheria vaccines (5). More recently, Endy et al. showed that antibody responses of individuals vaccinated with a purified chick embryo cell rabies vaccine, given on a postexposure prophylaxis schedule, were significantly lower in individuals receiving chloroquine compared with controls (6).

HY and chloroquine are lysosomotropic drugs that increase the pH of the lysosome, thus affecting functions of proteins involved in antigen presenting pathways and in B-cell activation (7). To the best of our knowledge, there are no new facts in the scientific and medical literature that indicate that the same mechanism could not operate in HY-treated patients suffering from COVID-19 and negatively impact their SARS-CoV-2-specific antibody responses. Indeed, recent findings indicate that some individuals, including hospitalized patients, who have recovered from COVID-19 have not made vigorous IgG antibody responses. However, the most comprehensive publications addressing antibody responses, wherein study subjects presented viability in levels of IgG antibody responses, have not detailed the treatment regimens delivered to the subjects (8–11).

## Discussion

Plans for employing immunity profiles against SARS-CoV-2 to relax social distancing and other epidemic mitigation measures and to create “immunity passports” to control spread of COVID-19 have recently been questioned by the World Health Organization because of uncertainty regarding antibody responses (12). As more needs to be learned about the role of antibodies in recovery from and protection against infection with SARS-CoV-2, the impact of HY and other treatment regimens on antibody responses requires systematic evaluation.

## Author Contributions

All authors listed have made a substantial, direct and intellectual contribution to the work, and approved it for publication.

## Conflict of Interest

The authors declare that the research was conducted in the absence of any commercial or financial relationships that could be construed as a potential conflict of interest.
