# Corrigendum: Impact of Hydroxychloroquine on Antibody Responses to the SARS-CoV-2 Coronavirus

**DOI:** 10.3389/fimmu.2020.615628

**Published:** 2020-11-02

**Authors:** Isabel Kinney Ferreira De Miranda Santos, Carlos Henrique Nery Costa

**Affiliations:** ^1^ Ribeirão Preto School of Medicine, University of São Paulo, São Paulo, Brazil; ^2^ Nathan Portela Institute of Tropical Medicine, Federal University of Piauí, Teresina, Brazil

**Keywords:** COVID-19, Sars-CoV-2, coronavirus, antibodies, hydroxychloroquine

In the original article, we neglected to include the funder Fundação de Amparo à Pesquisa do Estado de São Paulo (FAPESP), Grant Number 2020/09093-5 to IM.

The authors apologize for this error and state that this does not change the scientific conclusions of the article in any way. The original article has been updated.

